# Graduate Study in London

**Published:** 1908-02-15

**Authors:** 


### HOSPITAL ADMINISTRATION.

### CONSTRUCTION AND ECONOMICS.

### GRADUATE STUDY IN LONDON.

I.—THE POLYCLINIC AND ITS WORK.

Graduate study, so far as the medical profession is concerned, is by no means a novelty in England, but it has never attracted the attention or received the consideration of the profession to the extent which it has in America, in Germany, in Austria, at St. Petersburg and elsewhere. So little, indeed, has it been deemed worthy of serious thought that most of the primary attempts to interest the general practitioner in what is familiarly styled "post-graduate work," have proved abortive. A few special classes, held with a specific object in view, gained a measure of temporary success, but the peculiar circumstances in which they were initiated and the fact that they were designed to meet the necessities of a section of the profession at a time of exceptional strain, made them unique examples, the success of which did not encourage the profession generally to support schemes of graduate study. Gradually, however, we have come to see that there is a large amount of solid good underlying this "presumptuous impertinence" (as a former President of the Royal College defined it) that a qualified and duly licensed practitioner has anything to learn about his profession. The Polyclinic is a splendid reality to-day, and elsewhere, in London and in the other centres of medical education in England, there is a praiseworthy endeavour to emulate its success and to follow its lines.

In a former issue of The Hospital [April 20, 1907, p. 91] we gave an account of the institution in Chenies Street, and there is no necessity here to repeat the details then given, but a brief outline of the work, or rather of its scope and objects, which the institution has so magnificently done for the last ten years will be instructive. We say "magnificently" advisedly. A pioneering attempt must always command a large amount of respect and admiration, and when it has surmounted such obstacles and brought forth such excellent results, it is permissible to assert that it has been more than usually good, more than ordinarily effective. No one who takes the trouble to consider the history of the Polyclinic can doubt that it has achieved grand results. It started unobtrusively more than nine years ago (to be exact, in May 1899): it drew attention, not because it advertised in the press, but because it put forward its claims by results, because it had in every member a standing advertisement, an eager, proselytising disciple, keen as St. Pancras for the conversion of his immediate patriarchate. In the graduate-study movements facts count for more than mere figures. Put it blandly to the practitioner that he can obtain certain privileges and certain desirable advantages if he adopts a particular course that demands a modicum of personal inconvenience—and you will achieve little. Show him that there are brother practitioners who have done this, who have gone back to the work, who have manifestly benefited by it, and you will gain much. This latter course is the one that the Polyclinic has followed. Every year it betters itself—a proof of vitality: every term it increases its clientele—a sure mark of virility. To-day it offers to the graduated medical student advantages which no other institution in the Empire can equal.

Consider for a moment what these advantages are. "Remember," said Mr. Balfour at the Polyclinic's festival dinner in 1901, "remember what is the life of a general practitioner in a general practice. It is a hard day-to-day and night-to-night struggle with disease: no certainty of repose, no habitual opportunity of study, constant aid to the poor. Now to these hardworked and overworked general practitioners comes the duty of attempting to make themselves familiar with the latest researches in medical science, the accumulated wealth of medical experience, the vast mass of information contained in medical and other scientific journals concerning the latest results of medical investigation. . . . It cannot possibly be done under existing conditions. The Polyclinic has set itself to work to give these men, in their rare opportunities of leisure, on the easiest and cheapest terms, an opportunity of keeping abreast with the latest developments of medical science." It acquired, shortly after its inception, the premises in Chenies Street, W.C., formerly a commodious high school, and it adapted these to its needs, and has since enlarged and modified them. It gives the medical graduate the comforts of a club with the advantages of personal tuition if desired, of facilities for independent work, for coming into contact with leaders of medical thought, and for making special investigations. On five days of the week special cliniques are held in the large clinical hall. Here selected cases are shown and discussed, as in hospital out-patient departments—with this difference, that at out-patients the student may waste time in seeing, one after the other, patients who suffer from the same complaint, whereas here he sees selected cases only. Not that he necessarily sees "obscure" cases merely. The Polyclinic does not set out to teach the abstruse. Its aims are practical, not pyrotechnical; it concerns itself more with treatment than with theory of causation. Therefore, at these clinical demonstrations, which are, as they deserve to be, exceedingly popular, cases of common complaints are shown as well as baffling conditions in which the elucidation of the diagnosis is attended with more than ordinary difficulties. The clinics are therefore a conservation of energy—conservation for the men who are not asked to give attention to recurrent conditions, and conservation to the teachers who are not demanded to repeat their demonstrations an indefinite number of times every afternoon. On an average 1,200 different cases are shown at these demonstrations yearly.

Lectures on medicine, surgery, and allied subjects are delivered daily, except on Fridays and Saturdays. At these lectures the subject is treated not from the point of view of the unqualified student, but from that of a criticising, discriminating general practitioner. The fee payable for the general work of the College is an extremely modest one. For the annual payment of one guinea registered medical practitioners of either sex acquire the following privileges:—

1. Use of library, museum and reading-rooms.
2. The right of attendance at all cliniques and lectures.
3. The right of bringing or sending patients for consultatory advice.
4. The receipt of the College journal monthly.
5. The examination of pathological specimens at special fees.

There is a large and well-lit and exceptionally well stocked pathological and bacteriological laboratory, and practical classes are held daily in various subjects. The list of subjects dealt with during the present term at these classes serves very well to show the catholicity of the instruction given and the wide range which the curriculum covers. The list is as follows: Clinical microscopy (twice weekly), clinical examination of the nervous system (once a week), practical gynæcology (twice a week), *x*-ray work (once a week—the institution possesses a fine installation of apparatus for this work), applied anatomy (twice weekly), practical otology, practical laryngology, and practical ophthalmology (classes are held once a week in each of these subjects). The fees for these classes are exceptionally low; in fact, the Polyclinic is the cheapest place for medical study in London. The composition fee for attending all these courses is seven guineas; the fee for each class one guinea, excepting practical gynæcology, for which two guineas are charged.

In addition, the institution does a large amount of excellent tutorial work. The tutorial classes are destined for men working for special and higher examinations, and individual attention is given to each candidate by tutors who have had a large amount of experience in coaching work. The success of this department has been most encouraging, and in "number of passes" and "standard of passes" the Polyclinic stands well in the front rank of tutorial establishments in London and elsewhere. The composition fee for fifteen weeks' tuition in four subjects (medicine, surgery, midwifery, and pathology) is £15 15s., but any of these subjects can be taken separately. Thus the candidate working for his final Fellowship examination can do all his work at the Polyclinic at a cost 25 per cent. below that which such work entails at any other metropolitan institution. Special arrangements have been made whereby such candidates attend a class in operative surgery outside the institution and for facilities to obtain a first-hand knowledge of surgical treatment in the wards of a large hospital. The social advantages of the institution such as the Polyclinic need not be elaborated, but the club side of the establishment is as well looked after, as the teaching part. A special feature is the fine smoking-room, which is also used as a reference-room. Here are kept a classified series of references, cuttings from medical papers on every conceivable subject properly indexed and of incalculable usefulness to candidates at work on a thesis. Close at hand is the library, which we venture to think is one of the most excellent practitioners' libraries to be met with.

What is the success of the work? Gradually, as the institution is becoming better known, its clientele is increasing, as a glance at the attendance-book will show. Here are the figures: Attendances—1903, 14,538; 1905, 14,891; 1907, 14,950. The number of practitioners who attend has also increased progressively. It now averages 1,000 yearly. The average number of practitioners attending the special and tutorial classes is 250—a number which is steadily rising—and the average number of pathological investigations carried on annually is 600. These figures speak eloquently in favour of the rapidly increasing popularity of the institution, and they show conclusively that graduate study in England is rapidly winning favour with the profession. It is only by actual work, practical work at the bedside or in the laboratory that the practitioner can gain the fullest measure of experience, that he can effectively recognise what he has yet to learn, and so plan his course as best to ensure the maximum of efficiency with the minimum waste of energy. There is at present a tendency in the United States to encourage "graduate teaching by correspondence," a scheme which is doomed to failure, since it lacks the very essentials that count for success in such work—individual attention, a careful consideration of each man's limitations, in time, in expenditure, in capacity, and finally in choice of subject. Here at the Polyclinic that is the main object which is ever held in view. The medical school of to-day unfortunately has its eye on the examination lists. It is becoming a cramming establishment, in which the desirability of passing in the shortest possible time is held out to the pupil as an implied, if not actually admitted, "final end" superior to really thorough grounding in essentials. At the Polyclinic, where examinations count for far less, the practical value of the work is placed in the forefront and the practitioner realises how greatly he may gain by utilising in his every-day work the newest helps to diagnosis, treatment, and prophylaxis that modern science and research have given him.

In a future article we hope to deal with the limitations of the Polyclinic as the type of British post-graduate institutions and to consider in what way and by what means its effectiveness as an educative force may be increased, widened, and augmented by further organisation and endeavour.

